# A High-Performance Micro Differential Pressure Sensor

**DOI:** 10.3390/mi15111396

**Published:** 2024-11-20

**Authors:** Xutao Fan, Lei Wang, Songsong Zhang

**Affiliations:** 1 School of Microelectronics, Shanghai University, Shanghai 201800, China; fanxutao@shu.edu.cn; 2 Chengdu Chimesen Tech. Inc., Chengdu 610000, China; wanglei@chimesen.com; 3 Shanghai Melon Tech. Inc., Shanghai 201899, China; 4 Shanghai Industrial μTechnology Research Institute, Shanghai 201899, China

**Keywords:** micro electromechanical system (MEMS), micro differential pressure sensor (MDPS), silicon-on-insulator (SOI), small dimension

## Abstract

With the development of the micro electromechanical system (MEMS), which widely adopts micro differential pressure sensors (MDPSs), the demand for high-performance MDPSs had continuously increased. Pressure sensors realized using MEMS technology integrated with biomedical catheters are of significant importance in the detection and treatment of various biological diseases. Biomedical catheters used in low-Fr applications (1Fr = 0.33 mm outer diameter) require miniaturized sensors that do not compromise their performance. For instance, catheters (5Fr) used for central venous pressure (CVP) monitoring require the integration of high-performance sensors with total dimensions smaller than 1.65 mm along at least two directions (length, width, or height). In this paper, a silicon-on-insulator (SOI)-based MDPS was designed and fabricated for micro-pressure detection in the range of 0–1 kPa. The dimension of the sensor is only 1 mm × 1 mm × 0.4 mm, with a sensitivity of 3.401 mV/V/kPa at room temperature, nonlinearity of 0.376% FS (full scale), and an overall accuracy of 0.59% FS. The sensor operates normally when the temperature is even increased to 160 °C, and its temperature coefficient of zero output (TCO) and temperature coefficient of sensitivity (TCS) are 0.093% FS/°C and −0.144% FS/°C. The dimension and performance results of this MDPS demonstrate its potential to play a significant role in biomedical catheters. In addition, it is fabricated using an 8-inch MEMS process, which significantly reduces the cost.

## 1. Introduction

Pressure sensors can monitor and control various physical parameters such as pressure and flow across different measurement ranges [1]. Compared to generic pressure sensors, micro differential pressure sensors (MDPSs) typically have a measurement range of only a few kPa or even a few hundred Pa; this imposes stricter demands on the sensitivity and accuracy of the sensors. The MDPS focuses on detecting minute pressure differences, making it suitable for applications that are sensitive to pressure variations, while the generic pressure sensor is primarily used for measuring absolute or relative pressure. For the ultra-low pressure ranges (0.5–3 kPa), the average sensitivity of the pressure sensor is 4–5 mV/V/kPa, and to meet this requirement, a pressure sensor with an area of 10–25 mm^2^ and a profiled diaphragm with a thickness W of the thin part in the 8–12 μm range or below is required [2].

In various biomedical devices, catheters play a critical role in many medical procedures such as angioplasty [3], angiogram [4], ablation [5], minimally invasive surgery [6], neonatal care [7], and urology [8]. With advances in micro electromechanical systems (MEMS) technology, numerous miniature sensors have been developed as integral components of catheters. Central venous pressure (CVP) monitoring [9] provides essential physiological information in critical care, postoperative management, and fluid therapy, helping to improve patient outcomes and treatment effectiveness. Catheters used for CVP measurement belong to a class of intravascular catheters, typically available in dual-lumen (5 Fr/7 Fr/8 Fr), triple-lumen (5.5 Fr/7 Fr/8.5 Fr), and quadruple-lumen (8.5 Fr) sizes, where 1 Fr represents a catheter outer diameter of 0.3 mm [10]. This places strict requirements on sensor dimensions for compatibility. Capacitive MDPSs are limited in their application in low-pressure environments due to their large outer diameter and significant nonlinearity [11]. Piezoresistive pressure sensors have become the favored choice for micro differential pressure applications, owing to their compact dimension, ease of integration, good linearity, and cost efficiency [12,13,14]. Furthermore, advanced manufacturing techniques enable the miniaturization of piezoresistive pressure sensors. For instance, deep reactive ion etching (DRIE) [15] is employed to create deep and narrow features in the silicon wafer, thereby reducing the silicon area. Ion implantation [16] is utilized for precise doping control, enhancing the reliability of the sensors. However, in all pressure ranges, there is a trade-off between increasing sensitivity and reducing dimension. To enhance sensitivity while maintaining or reducing the chip dimension, it is essential to decrease the diaphragm thickness, but this inevitably leads to increased nonlinearity. Furthermore, piezoresistive pressure sensors are sensitive to temperature changes, resulting in a temperature coefficient of zero output (TCO) and a temperature coefficient of sensitivity (TCS) in the output signal [17], which lowers accuracy over a wide temperature range [18,19]. Research has shown [20,21,22,23,24,25] that silicon-on-insulator (SOI) technology enables pressure sensors to function at higher ambient temperatures and allows for better control of diaphragm thickness.

In recent years, significant achievements have been achieved in the design and fabrication of MDPSs. Yu et al. improved the traditional convex diaphragm by adding a beam to focus stress near it, resulting in a notable improvement in sensitivity, achieving 11.098 mV/V/kPa over the 0–500 Pa range, with only 3.046% FS nonlinearity [26]. Chuang Li et al. designed a piezoresistive micro pressure sensor with a four-slotted diaphragm and beam, fabricated on SOI wafers using micro electro mechanical system (MEMS) micromachining and anode-bonding techniques. This sensor achieved 4.48 mV/V/kPa sensitivity at 6.89 kPa [27]. Mikhail Basov’s research on a pressure sensor for ultra-low differential pressure environments yielded a device measuring 6.15 mm × 6.15 mm, with an average sensitivity of 34.5 mV/V/kPa and nonlinearity of 0.81% FS [28].

Most research to date has focused on optimizing individual performance factors, such as sensitivity, which frequently results in poor linearity, low accuracy, and increased dimension. In particular, excessive dimensions hinder miniaturization, limiting applications in fields such as biomedicine. This study systematically examines the operational principles and root causes of temperature drift in piezoresistive pressure sensors. By optimizing the manufacturing process and conducting device simulations, we achieve a balance between sensitivity and TCS. The sensors were fabricated using an 8-inch MEMS process, with performance evaluation conducted after basic packaging. The sensor’s dimension of only 1 mm × 1 mm highlights its strong potential for CVP monitoring applications.

## 2. Theory, Design, and Simulation

### 2.1. Sensor Principle and Temperature Drift Inducements

**Figure 1:**
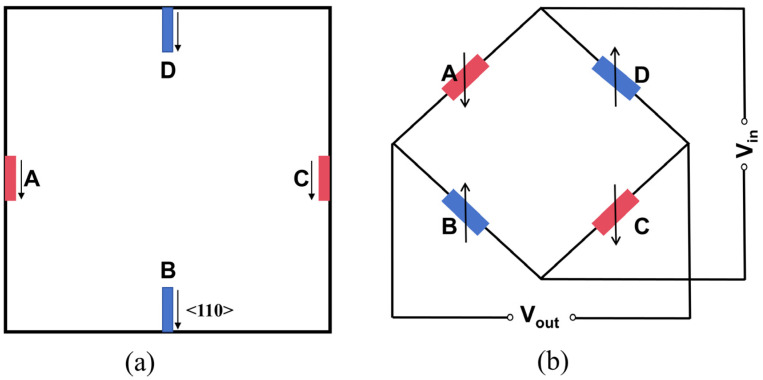
(**a**) Schematic diagram of a typical P-type piezoresistive resistor. (**b**) Wheatstone bridge diagram.

The piezoresistive effect refers to the change in the resistivity of a semiconductor when subjected to mechanical stress, resulting from shifts in energy bands and the movement of energy valleys. This phenomenon was first observed by Smith in 1954 during investigations into the resistance and stress characteristics of silicon and germanium [29]. Piezoresistive pressure sensors leverage this effect within resistors. As illustrated in Figure 1a, a typical design incorporates P-type piezoresistors symmetrically arranged along the edges of a square diaphragm. When pressure is applied to the diaphragm, the stress on the piezoresistors acts perpendicular to the edges and directs toward the diaphragm’s center. This results in a reduction in the resistance of *A* and *C*, while *B* and *D* experience an increase in resistance. By connecting the four piezoresistors in a Wheatstone bridge arrangement [30], as shown in Figure 1b, the stress-induced changes in resistance create a bridge imbalance, yielding a differential output. This output can be expressed as follows:

(1) $$V_{out}=V_{in}\left(\frac{R_{B}}{R_{A}+R_{B}}-\frac{R_{C}}{R_{C}+R_{D}}\right)$$

where *V_out_* and *V_in_* denote the output voltage and excitation voltage, respectively. Under ideal conditions, *R_A_* = *R_B_* = *R_C_* = *R_D_* = *R*, the sensor output can be formulated as follows:

(2) $$V_{out}=V_{in}\frac{∆R}{R}$$

From Equation (2), it can be seen that the sensor’s output is contingent upon the change in resistance [31]. The fractional change in resistance can be expressed as follows:

(3) $$\begin{array}{c} \frac{∆R}{R}=\pi_{l}\sigma_{l}+\pi_{t}\sigma_{t} \end{array}$$

where *π_l_* and *π_t_* represent the longitudinal and transverse piezoresistive coefficients, while *σ_l_* and *σ_t_* represent the longitudinal and transverse stresses on the piezoresistive resistor. Single-crystal silicon has three independent piezoresistive tensor coefficients *π*_11_, *π*_12_, and *π*_44_ [32], corresponding to the longitudinal, transverse, and shear piezoresistive coefficients in the standard coordinate system. Therefore, for any *π_l_* and *π_t_*, they can be expressed in terms of *π*_11_, *π*_12_, and *π*_44_. For <110> orientation P-type silicon, the *π*_11_ and *π*_12_ terms can be neglected compared to *π*_44_. Thus, Equation (2) can be rewritten as follows:

(4) $$V_{out}=\frac{1}{2}\pi_{44}\left(\sigma_{l}-\sigma_{t}\right)V_{in}$$

From Equation (4), it can be observed that when the sensor is subjected to zero pressure, the sensor’s output is zero.

However, temperature fluctuations can lead to changes in resistance, which subsequently cause the sensor’s offset voltage to vary with temperature. The effect of temperature on resistance can be expressed as follows:

(5) $$R\left(T\right)=R_{0}\left(1+\alpha ∆T+\beta ∆T^{2}\right)$$

where *R*(*T*) and *R*_0_ represent the resistance values at the operating temperature *T* and the reference temperature *T*_0_, respectively. Δ*T* denotes the temperature difference between the reference and operating conditions, while *α* and *β* are the temperature coefficients of resistance (TCR). The offset voltage caused by TCR can be expressed as follows [33]:

(6) $$V_{0}\left(T\right)=\frac{1}{4}V_{in}\sum_{1}^{4}{\left(-1\right)}^{i+1}\left(\alpha_{i}∆T+\beta_{i}{∆T}^{2}\right)$$

where *i* indicates the index of the piezoresistive resistor (*i* = *A*, *B*, *C*, *D*). The equation shows that when the quadratic term is zero, the zero-offset output is linearly dependent on temperature, either positively or negatively. When the quadratic term is non-zero, the zero-offset output exhibits a quadratic temperature dependence.

(7) $$\pi \left(N,T\right)=P\left(N,T\right)\pi \left(N_{0},300\;K\right)$$

where *π* (*N*, *T*) represents the piezoresistive coefficient at any doping concentration and temperature, *π* (*N*_0_, 300 K) is the piezoresistive coefficient under lightly doped conditions (<10^16^cm^−3^) at room temperature, and *P* (*N*, *T*) is an analytical expression that relates *π* (*N*, *T*) to *π* (*N*_0_, 300 K). Richter et al. proposed an updated model for the piezoresistive coefficients of P-type silicon as a function of doping concentration and temperature in 2008 [34]. This model has been simplified into an approximate analytical formula:

(8) $$P\left(N,T\right)=\frac{T_{n}-\theta }{1+\left(\frac{N}{N_{b}}\right)\alpha T_{n}-\beta +\left(\frac{N}{N_{c}}\right)\gamma T_{n}-\eta }$$

where *T_n_* = *T*/300 is the normalized temperature, and *N* is the doping concentration. The coefficients *θ*, *N_b_*, *N_c_*, *α*, *β*, *γ*, and *η* are fitting coefficients. The piezoresistive coefficients predicted by the Richter model are accurate across the full temperature range for lower doping concentrations, but they tend to be overestimated at higher doping levels. To improve the accuracy of the piezoresistor model, Joseph C. Doll et al. [35] corrected the Richter model in 2013, making the modified version accurate across the full range of temperatures and doping concentrations. The specific fitting coefficients can be found in that reference. This modified model will be utilized for calculating the piezoresistive coefficients in the subsequent sections of this paper. When the sensor’s operating temperature is *T*, substituting Equation (7) into Equation (4) and combining it with Equation (6) yields the full-bridge output voltage, which can be expressed as follows:

(9) $$V_{out}\left(T\right)=\frac{1}{4}V_{in}\sum_{1}^{4}{\left(-1\right)}^{i+1}\left(\alpha_{i}∆T+\beta_{i}{∆T}^{2}\right)+\frac{1}{2}\pi_{44}\left(N_{0},300\;K\right)P\left(N,T\right)\left(\sigma_{l}-\sigma_{t}\right)V_{in}$$

**Figure 2:**
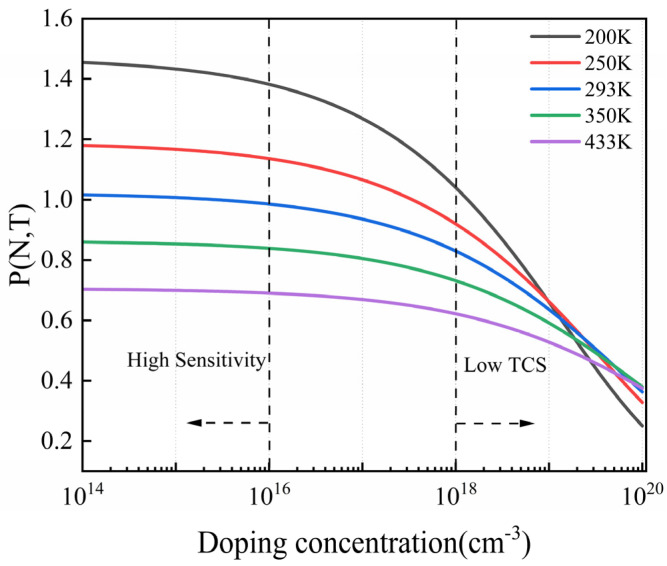
P (N, T) coefficient of P-type silicon vs. doping concentration for different temperatures.

The first term represents the change in zero output with temperature, while the second term reflects the change in the piezoresistive coefficient caused by temperature variations, which affects sensor sensitivity. To visualize the relationship between the piezoresistive coefficient, doping concentration, and temperature, Equation (8) is plotted in Figure 2. At low doping concentration (<10^16^ cm^−3^), the piezoresistive coefficient changes minimally dependent on doping concentration but is highly sensitive to temperature. Conversely, at high doping concentrations (>10^18^ cm^−3^), the piezoresistive coefficient is relatively unaffected by temperature, though it decreases rapidly, resulting in reduced sensitivity. Thus, TCS and sensitivity are in conflict.

### 2.2. Design and Simulation

**Figure 3:**
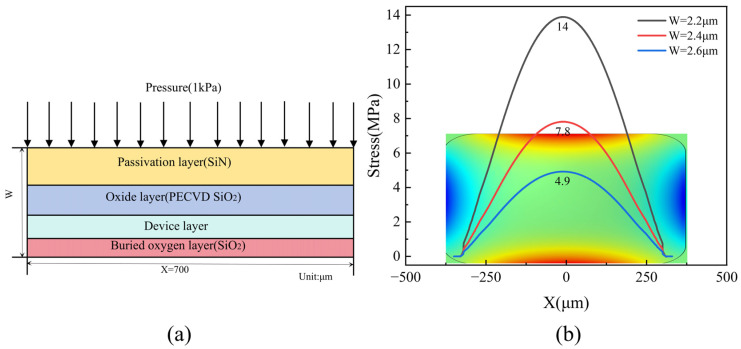
(**a**) Schematic diagram of inductive diaphragm. (**b**) Stress distribution on the surface of a piezoresistor at 10 μm from the edge of the diaphragm under an applied pressure of 1 kPa.

The pressure sensor is designed for an operating range of 0–1 kPa, using a conventional design that utilizes a (100)-type crystal, a square diaphragm, and P-type piezoresistors. All piezoresistors are arranged along the <110> crystal direction, as illustrated in Figure 1a. In consideration of process cost, packaging challenges, and market demand and based on the design guidelines in [36], we selected the sensor dimensions to be 1 mm in side length and 0.4 mm in thickness, with the sensing diaphragm measuring 0.7 mm × 0.7 mm. Given that diaphragm thickness significantly influences the sensor’s nonlinearity, we established a controllable range for diaphragm thickness and optimized it by changing the device layer thickness. According to the wafer manufacturer’s specifications, the insulating layer thickness is set to 0.4 μm. Figure 3b depicts the stress distribution at 10 μm from the diaphragm edge under an applied pressure of 1 kPa for different diaphragm thicknesses W. Notably, stress increases as the diaphragm thickness decreases, and, according to Equation (4), the stress magnitude is proportional to device output, thereby enhancing sensitivity.

The piezoresistor formed by ion implantation has a non-uniform distribution of doping concentration [37]. While the sensor’s sensitivity is prodominantly influenced by the surface doping concentration, the TCS is mainly determined by the bulk concentration [38]. Therefore, to enhance sensitivity while minimizing TCS, it is essential to maximize the bulk concentration of the piezoresistor and minimize the surface concentration. We observed that when the thickness of the device layer and the parameters of the ion implantation process are fixed, the bulk concentration of the piezoresistor remains constant. However, reducing the device layer thickness to match the original junction depth of the piezoresistor increases its average bulk concentration, thereby lowering the TCS. Thus, accurately calculating the optimal thickness of the device layer is critical.

**Figure 4:**
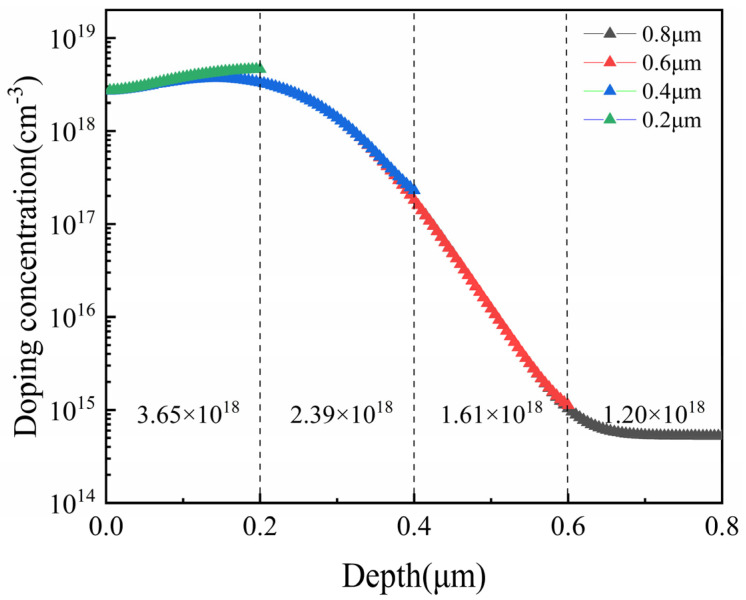
Distribution of doping concentration of the piezoresistor in the depth direction for different device layer thicknesses.

**Figure 5:**
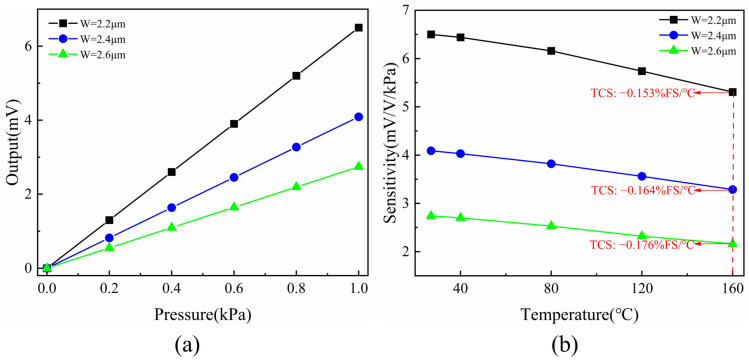
Simulation results for different diaphragm thicknesses W: (**a**) Output vs. Pressure. (**b**) Sensitivity vs. Temperature.

In the previous section, we examined the impact of doping concentration and temperature on the piezoresistive coefficient. For this study, the doping concentration of the piezoresistor was set to 1 × 10^18^ cm^−3^. The specific fabrication process parameters were obtained using a TCAD simulator (silvaco 2019), and after several adjustments to optimize the process, the key process parameters are summarized in Table 1. Using these parameters, we established the doping concentration distribution in the depth direction of the piezoresistor for different device layer thicknesses of the pressure sensor. As shown in Figure 4, the surface doping concentrations for different device layer thicknesses nearly coincide when the piezoresistor is formed by ion implantation. For device layer thickness of 0.6 μm and 0.8 μm, the depth distribution of the doping concentration remains nearly identical, with a junction depth of 0.59 μm, as extracted by the simulation software (silvaco 2019). For thicknesses of 0.2 μm and 0.4 μm, this corresponds to the thickness of the piezoresistor. At this point, we can calculate the doping concentration of piezoresistors formed at different device layer thicknesses. Substituting this value into Equation (8) allows us to determine the change in the piezoresistive coefficient when the temperature increases from room temperature (293 K) to 433 K. We can also estimate the device’s sensitivity using this value in Equation (8). As shown in Figure 5, the sensitivity of the pressure sensor increases while the TCS decreases as the diaphragm thickness decreases. We found that reducing the device layer thickness lowers the TCS to some extent due to the increased doping concentration of the piezoresistor. Although a lower piezoresistive coefficient diminishes sensitivity, the overall device sensitivity improves because the effect of stress is much greater than that of the piezoresistive coefficient. Thus, while we aim to minimize the thickness of the device layer, making the piezoresistor too thin may compromise device reliability and result in excessively high resistance. For this reason, we have selected a device layer thickness of 0.2 μm and a diaphragm thickness of 2.2 μm.

The square resistance of the pressure sensor was directly measured as 1034 Ω/□ for the piezoresistor with a device layer thickness of 0.2 μm, and 66 Ω/□ for the internal interconnect structure. The doping concentration distribution was obtained. We employed a method proposed by Sridhar and Foster to calculate the TCR using the doping concentration distribution [39]. This method treats the piezoresistor as a series of very thin resistor sheets stacked vertically, assuming uniform doping concentration within each sheet. The resistance of the piezoresistor can be expressed as follows:

(10) $$R\left(T\right)=\frac{1}{G\left(T\right)}$$

where *R*(*T*) represents the temperature-dependent resistance of the piezoresistor and *G*(*T*) represents its temperature-dependent conductance. According to this approach, *G*(*T*) is calculated by the following formula:

(11) $$G\left(T\right)=\sum_{i}q\mu_{p}\left(T,N_{toti}\right)P\left(T,N_{neti}\right)\left[D_{i-1}-D_{i}\right]$$

where *q* is the electron charge. *μ_p_* (*T*, *N_toti_*) is the hole mobility in the P-type piezoresistor, dependent on temperature and total doping concentration, *N_toti_*. *P* (*T*, *N_neti_*) is the hole concentration, which varies with temperature and net doping concentration, *N_neti_*. *D_i_*_−1_ − *D_i_* is the thickness of each thin resistor sheet. The detailed calculation methods are provided in the referenced literature. The final expression for the piezoresistor with respect to temperature was obtained by expanding *R*(*T*) in the form of Equation (5) using binomial expansion. The previously obtained doping concentration was brought into this equation, leading to numerically calculations that yielded a final resistance temperature coefficient of 1223 ppm/°C and a square resistance of 987 Ω/□ for the piezoresistor. The calculated square resistance closely aligns with the value obtained from TCAD simulations, indicating the accuracy of the simulation. At this time, the square resistance of the interconnect structure meets the requirement of less than 100 Ω/□, while the piezoresistor exhibits a low resistance temperature coefficient.

**Figure 6:**
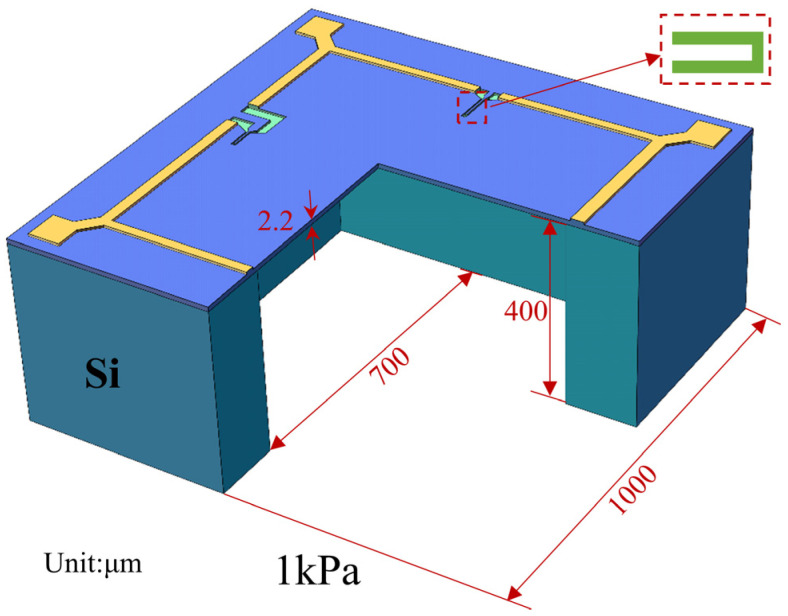
Three-dimensional model drawing of the sensor (values is μm).

In the design of the piezoresistor to improve the sensor’s sensitivity, it should be placed in the region of maximum stress concentration on the sensing diaphragm. Both the dimension and number of turns of the piezoresistor significantly affect sensitivity; larger dimensions can cause stress averaging, reducing the overall sensitivity. Therefore, the piezoresistor dimension must be appropriately controlled. This study adopts a bent piezoresistor with two turns, consisting of a piezoresistor with a planar size of approximately 2 μm × 30 μm and connecting arms spaced 4 μm apart. An intermediate heavily doped region facilitates the connection between the piezoresistor and the Wheatstone bridge electrodes, which are typically composed of heavily doped silicon or metal [40]. Figure 6 presents a schematic of the completed sensor design.

## 3. Manufacturing and Testing

**Figure 7:**
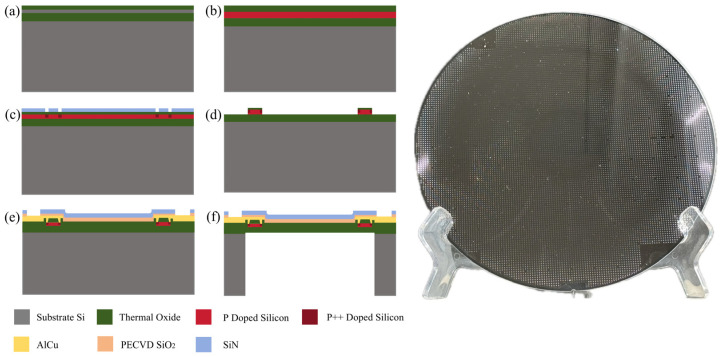
Manufacturing process flowchart and 8-inch MEMS wafer of the pressure sensor. (**a**) thermal oxidation; (**b**) boron light doping; (**c**) boron heave doping; (**d**) excess silicon; (**e**) deposition of silica and metal wiring; (**f**) back cavity release.

A resistive pressure sensor fabricated on an SOI wafer has been successfully completed, with the fabrication process illustrated and 8-inch MEMS wafer in Figure 7. In Figure 7a, the substrate consists of a 725 μm thick n-type (100) silicon wafer, which has a 0.4 μm thick buried oxide layer and a 0.2 μm thick device layer. The SOI wafer is subjected to a high-temperature oxidation furnace to grow a 20 nm thick layer of silicon dioxide. This thermal oxidation is performed to suppress ion channeling and to enhance the scattering probability during ion implantation. In Figure 7b, light doping of P ions is performed by implanting boron ions into the top layer of the wafer using ion implantation, with an implantation dose of 1 × 10^14^ Ion/cm^2^ and an energy of 40 keV. In Figure 7c, heavy doping of P ions (P++) is achieved by localized bulk doping of boron ions using photolithography and masking techniques, with an implantation dose of 1 × 10^15^ Ion/cm^2^ and an energy is 25 keV. Afterward, an annealing process at 1000 °C for 15 min in an oxidation furnace is carried out to repair any lattice damage. In Figure 7d, excess silicon and silicon dioxide are etched away, leaving only the piezoresistive resistor structure. In Figure 7e, A 0.4 μm thick silicon dioxide layer is prepared using Low-Pressure Chemical Vapor Deposition (LPCVD) of tetraethyl orthosilicate (TEOS). Photolithography and dry etching are performed on the front-side silicon dioxide layer to expose the heavily doped piezoresistive resistor and electrode contact areas. Due to the reduction in boron ions on the surface resulting from the previous annealing step, secondary doping is performed to create good ohmic contacts. This is followed by the growth of a 10 nm silica layer at the through holes, with another annealing step at 1000 °C for 15 min. Subsequently, aluminum-copper (AlCu) is deposited to a thickness of 1 μm for the electrode connection area and wiring distribution of the Wheatstone bridge, followed by annealing at 430 °C for 30 min to increase electrical conductivity [41]. A 0.4 μm silicon dioxide layer and 1 μm silicon nitride (SiN) layer is grown using Plasma-Enhanced Chemical Vapor Deposition (PECVD). Electrode pads are formed through photolithography and etching processes. In Figure 7f, the wafer is thinned to 400 μm, and (DRIE) is performed to release the diaphragm structure.

**Figure 8:**
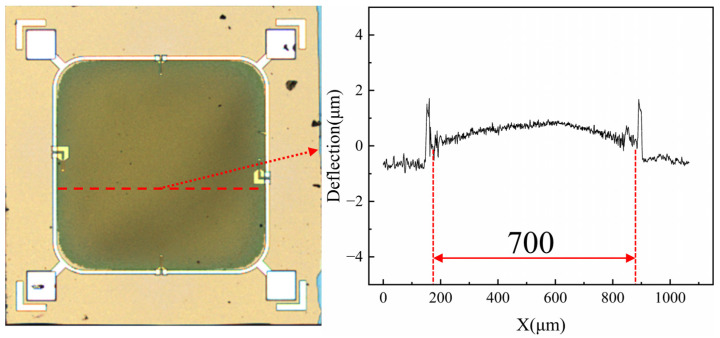
Profiler result plot of the pressure sensor.

Residual stresses in the film greatly affect the performance of pressure sensors [42]. As these stresses are unavoidable during the manufacturing and encapsulation processes, it is essential to select pressure sensors that exhibit minimal deflection for encapsulation and testing. The entire wafer was examined using a profiler, and Figure 8 shows the morphology of the pressure sensor as observed under the profiler. The sensor with the flattest film was selected for further testing.

**Figure 9:**
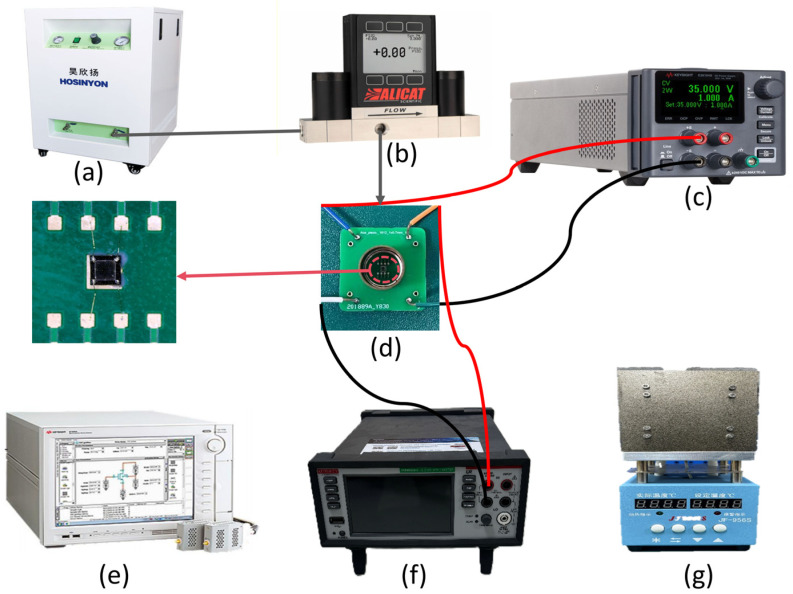
Diagram of the pressure sensor test environment. (**a**) gas pressure source; (**b**) pressure controller; (**c**) DC power analyzer; (**d**) test sample; (**e**) semiconductor device analyzer; (**f**) digital multimeter; (**g**) temperature-controlled heating stage.

To complete device testing, a simple assembly (packaging) method was adopted in this work. First, DELO BS3770 adhesive (manufactured by DELO Industrial Adhesives, located in Windach, Germany), a modified polyurethane derivative known for its high-temperature resistance and strong adhesion properties, was utilized to establish a soft connection between the chip and the Process Control Block (PCB). With a tensile strength of two megapascals, this adhesive satisfies the fixation and stress requirements while minimizing the stress caused by encapsulation. The device electrode and PCB electrode were connected with a metal lead, which was subsequently welded to the metal tube shell to ensure a stable connection with the PCB. An insulating glue was applied for further sealing. The final packaged device is shown in Figure 9.

The performance of the fabricated pressure sensor was tested using the setup shown in Figure 9. Gas pressure was provided by a pressure tank and delivered through a pipe to the sensor’s package housing. A flowmeter-based pressure standard was installed midway in the pipe to facilitate accurate control and monitoring of the input pressure. We employed the ALCAT pressure standard due to its high precision, which minimizes measurement errors and features a fast response time of 30 ms. A 1 V supply voltage was provided by a DC power source, and the output voltage of the sensor was measured and logged with a digital multimeter.

**Figure 10:**
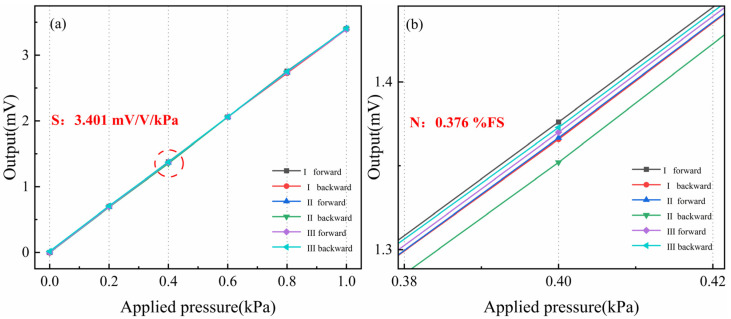
(**a**) Static performance test chart of the sensor. (**b**) Enlargement of detail from Figure 10a.

**Figure 11:**
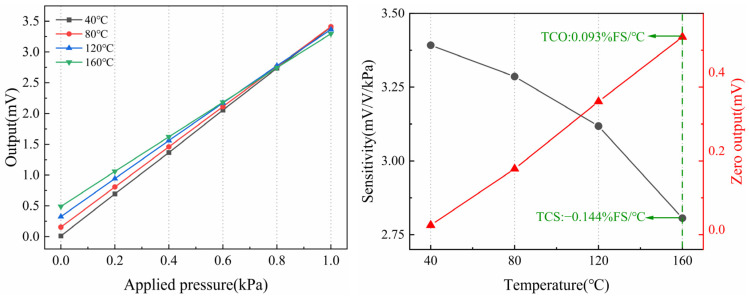
Temperature performance of the sensor.

As shown in Figure 10a, the pressure was loaded and unloaded in increments of 0.2 kPa, and the pressure values stabilized without further fluctuation. The pressure was smoothly increased to 1 kPa under a constant supply voltage of 1 V. After the values at each test point stabilized, the output from the pressure-sensitive element was recorded, and the load was gradually reduced. The sensitivity, linearity, repeatability, and hysteresis of the pressure sensor at room temperature were calculated to be 3.401 mV/V/kPa, 0.376% FS, 0.432% FS, and 0.157% FS. Compared with the simulation results, the lower-than-expected sensitivity is attributed to the depth of the varistor, leading to reduced actual stress, especially at the ends, where the stress is lower than in the middle. This discrepancy is not due to measurement errors. A temperature performance test was then conducted by gradually increasing the temperature from 40 °C to 160 °C in steps of 40 °C. As shown in Figure 11, the pressure sensor maintained good linearity up to 160 °C. With increasing temperature, the pressure sensor exhibited a TCS of −0.144% FS/°C, primarily due to the reduced piezoresistive coefficient at higher temperatures. The TCO was calculated to be 0.093% FS/°C, largely caused by the manufacturing process, which could not ensure that the four piezoresistors had identical properties. Compared to the simulation results, the sensor’s temperature drift is slightly smaller, which may be caused by some degree of temperature error.

Table 2 provides a comparison between the MDPS designed in this paper and related piezoresistive sensors. While the differences in operating ranges are minor, sensors with similar sensitivity and nonlinearity to this design are typically much larger. Sensors with much higher sensitivity are even bigger and tend to exhibit higher nonlinearity, resulting in lower accuracy. Compared to commercial sensors, the sensor in this paper demonstrates advantages in both sensitivity and temperature stability. Therefore, this sensor excels with its compact dimension, enhanced accuracy, and low-temperature drift, making it ideal for applications with stringent dimension constraints and high-temperature operation.

This study explores the feasibility of applying the fabricated pressure sensor for CVP monitoring. Benefiting from advanced manufacturing techniques, such as DRIE to reduce silicon area, SOI substrates for precise control over diaphragm thickness to achieve high sensitivity, and ion implantation for accurate doping control to enhance device reliability, a miniaturized piezoresistive pressure sensor has been developed. This sensor can be placed within a central venous catheter. In monitoring applications, a CVP reading below 0.49 kPa in shock patients indicates insufficient blood volume, requiring prompt fluid replacement. A CVP above 0.98 kPa, on the other hand, suggests excessive vasoconstriction or potential heart failure, necessitating controlled infusion rates or other interventions. The pressure sensor developed in this work, with a working range of 0–1 kPa, high sensitivity, and accuracy, meets the requirements for effective CVP monitoring.

## 4. Conclusions

In this paper, a high-performance MDPS has been successfully designed and fabricated. The sensor possesses a high sensitivity of 3.401 mV/V/kPa, a nonlinearity of 0.376% FS, and a combined accuracy of 0.59% FS in the range of 0–1 kPa. The TCO and TCS of the sensor were 0.093% FS/°C and −0.144% FS/°C, respectively, when the temperature was increased to 160 °C. And, the size of the sensor was only 1 mm × 1 mm. Both the simulation and experimental results demonstrate that the piezoresistive junction depth and doping concentration distribution can be modified by designing the diaphragm, resulting in improved temperature stability of the pressure sensor. The sensor offers advantages such as a small dimension, strong overall performance, and excellent potential for miniaturization. This study not only provides valuable insights into the miniaturization of high-performance MEMS sensors but also demonstrates the significant potential of the developed MDPS for CVP monitoring. This work was conducted using an 8-inch process, with a focus on individual device performance. While wafer-level data, including process margins, are important for understanding the full variability, this study does not include wafer-wide measurements. Future work will address the process margin variations to provide a more comprehensive understanding of the performance across the entire wafer.

## Figures and Tables

**Table 1:** Key process parameter.

Process	Parameter	
Light doping	Dose 1 × 10^14^ Ion/cm^2^	Energy 40 keV
Heavy doping	Dose 1 × 10^15^ Ion/cm^2^	Energy 25 keV
Anneal	Temperature 1000 °C	Time 15 min

**Table 2:** Comparison with other piezoresistive sensors.

Pressure Range (kPa)	Sensor Dimension (mm)	Sensitivity (mV/V/kPa)	TCS (% FS/°C)	TCO (% FS/°C)	Nonlinearity (% FS)	Accuracy (% FS)
0–1 [this work]	1 × 1	3.401	−0.144	0.093	0.376	0.59
0–0.5 [14]	7 × 7	11.089			3.046	3.204
0–6.8 [15]	3.6 × 3.6	4.48	−0.15	1.8	0.25	0.34
0–0.5 [16]	6.15 × 6.15	34.2	−0.280		0.81	
0–30 [36]	1 × 1	2.25	−0.221	−0.209		
0–3 [43]	3.4 × 3.4	4.67			0.18	
0–6 (Honeywell TSC Series)		2.6	±2	±1.15		

## Data Availability

The original contributions presented in the study are included in the article, further inquiries can be directed to the corresponding author.
